# Expression and prognostic significance of INSM1 compared with traditional neuroendocrine markers in mixed urothelial and small-cell carcinoma of the renal pelvis

**DOI:** 10.3389/fonc.2026.1861172

**Published:** 2026-07-20

**Authors:** Ji Li, Liang Cheng, Lunbi Wu

**Affiliations:** 1 Clinical Pathology Diagnosis Center, The Second Affiliated Hospital of Qiqihar Medical University, Qiqihar, China; 2 Department of Urology, The First Affiliated Hospital of Jiamusi University, Jiamusi, China

**Keywords:** immunohistochemistry, INSM1, neuroendocrine markers, renal pelvis, small-cell carcinoma, upper tract urothelial carcinoma

## Abstract

**Background:**

Mixed urothelial carcinoma (UC) and small-cell carcinoma (SmCC) of the renal pelvis is a rare but clinically consequential malignancy in which recognition of the neuroendocrine component directly influences diagnosis and treatment. We compared insulinoma-associated protein 1 (INSM1) with traditional neuroendocrine markers and explored clinicopathological correlates of outcome in a retrospective cohort of mixed renal pelvic UC/SmCC.

**Methods:**

Twenty-seven consecutive patients with histologically confirmed mixed renal pelvic UC/SmCC were identified from the institutional pathology archive during a prespecified consecutive 5-year accrual window (January 2019 to December 2023). Paired SmCC and urothelial compartments were evaluated separately on 4-μm formalin-fixed paraffin-embedded sections for INSM1, synaptophysin, chromogranin A, CD56, CK7, GATA3, Ki-67, and p53, using a modified H-score (0–300). Paired marker positivity was compared with the McNemar test, paired quantitative differences with the Wilcoxon signed-rank test, and overall survival was modeled with Kaplan–Meier estimation and Cox proportional hazards regression.

**Results:**

INSM1 showed the highest sensitivity for the SmCC compartment, with nuclear positivity in 26 of 27 cases (96.3%; 95% CI, 81.0–99.9%), compared with synaptophysin 59.3%, chromogranin A 18.5%, and CD56 85.2%; all paired UC components were INSM1-negative. INSM1 was significantly more sensitive than synaptophysin (P < 0.01) and chromogranin A (P < 0.001), whereas CD56 did not differ significantly from INSM1 (P = 0.375). CK7 and GATA3 were retained exclusively in the UC compartment, and the median Ki-67 index was markedly higher in SmCC than UC (76% vs 24%; P < 0.001). On univariable Cox analysis, advanced pT stage, nodal metastasis, predominant SmCC burden, and higher INSM1 H-score were adversely associated with overall survival, whereas adjuvant chemotherapy was associated with reduced mortality risk.

**Conclusions:**

INSM1 is a sensitive and compartment-specific marker for identification of the SmCC component and showed higher sensitivity than synaptophysin and chromogranin A in this cohort, while performing similarly to CD56. Combined with CK7 and GATA3, INSM1 helps delineate the urothelial and neuroendocrine lineages within a single biphasic tumor. Larger multi-institutional cohorts with molecular profiling are needed to validate these exploratory findings and to clarify whether quantitative INSM1 expression carries prognostic relevance.

## Introduction

Upper tract urothelial carcinoma (UTUC) accounts for approximately 5–10% of urothelial malignancies, yet it represents a biologically diverse and clinically consequential group of tumors (1, 2). In contrast to the bladder, where experience with variant histology is broader, tumors of the renal pelvis and ureter are encountered less frequently and are therefore more susceptible to under-recognition and under-reporting of unusual morphological patterns. This distinction has substantive implications, because variant histology in UTUC is not merely descriptive: contemporary meta-analyses and large single-institution series have consistently linked it to more advanced pathological stage, inferior oncological outcomes, and altered therapeutic decision-making relative to pure urothelial carcinoma (3–7). For practicing pathologists and urological oncologists alike, the priority is not only accurate identification of a rare variant but also recognition of the therapeutic implications that extend beyond standard UTUC management.

Among the rarest and most clinically consequential of these variants is small-cell carcinoma (SmCC), a poorly differentiated neuroendocrine carcinoma characterized by rapid proliferation, early metastatic dissemination, and a treatment logic that more closely follows extrapulmonary or pulmonary small-cell paradigms than conventional urothelial pathways (8, 9). Primary SmCC of the renal pelvis is itself exceedingly uncommon, and the mixed form, in which SmCC coexists with a urothelial carcinoma (UC) component, is rarer still. The published literature consists largely of case reports, small institutional series, and pooled reviews (10–15), but these sources deliver a consistent message: tumors in this category behave aggressively, often present at an advanced stage, and require precise histopathological recognition. Moreover, the frequent coexistence of conventional UC and SmCC carries biological implications. Integrated molecular studies in urinary bladder small-cell carcinoma have demonstrated shared genomic alterations between the urothelial and neuroendocrine components, including near-uniform TP53 and RB1 pathway disruption and evidence of urothelial-to-neural lineage plasticity, supporting a common clonal origin with divergent differentiation rather than a collision of unrelated neoplasms (16–19).

From a diagnostic standpoint, mixed renal pelvic UC/SmCC presents a distinctive challenge. In a generously sampled nephroureterectomy specimen with classic morphology, the neuroendocrine component is often recognizable on routine hematoxylin and eosin sections. In daily practice, however, tissue may be limited, necrosis may be extensive, and transition zones between urothelial and small-cell elements may be subtle. Traditional neuroendocrine markers, particularly synaptophysin, chromogranin A, and CD56, remain useful, but their performance is neither uniform nor entirely reliable in high-grade urinary tract tumors (20–23). Focal staining, weak labeling, crush artifact, and interpretive background can all obscure the diagnosis precisely when accuracy is most critical.

Insulinoma-associated protein 1 (INSM1) has emerged as an attractive marker in this setting. Unlike conventional cytoplasmic or membranous neuroendocrine markers, INSM1 is a nuclear transcription factor intrinsic to neuroendocrine differentiation, and this biological property confers interpretive advantages (20, 24, 25). Thoracic pathology studies, including a recent systematic review and meta-analysis, established a pooled sensitivity of 0.86 and specificity of 0.97 for pulmonary neuroendocrine carcinomas (26), while subsequent genitourinary investigations indicated that it retains strong diagnostic value in urinary tract and broader genitourinary high-grade neuroendocrine carcinomas (20, 21, 25). Equally important, nuclear labeling is often easier to evaluate in necrotic or crushed tissue, where cytoplasmic stains may be equivocal. What remains insufficiently defined is how INSM1 performs in the specific setting of mixed renal pelvic UC/SmCC, where the pathologist must not only confirm neuroendocrine differentiation but also delineate its spatial extent within a biphasic tumor.

The present study was designed to address that gap. Within a retrospective cohort of 27 mixed renal pelvic UC/SmCC cases, we compared the sensitivity and compartmental staining characteristics of INSM1 with synaptophysin, chromogranin A, and CD56; examined the reciprocal immunophenotypic features of the SmCC and UC components; and explored whether component burden and immunohistochemical variables were associated with overall survival. The objective was to provide a morphology-anchored and diagnostically practical analysis that reflects routine surgical pathology practice in this uncommon malignancy.

## Materials and methods

### Study design and case selection

This retrospective observational cohort study was conducted in accordance with the Strengthening the Reporting of Observational Studies in Epidemiology (STROBE) statement (27). The study setting was the Clinical Pathology Diagnosis Center of the Second Affiliated Hospital of Qiqihar Medical University. Eligible cases were identified from a consecutive 5-year interval between January 2019 and December 2023, corresponding to the period during which all nephroureterectomy specimens bearing a diagnosis of small-cell or neuroendocrine carcinoma of the upper urinary tract were continuously catalogued in the institutional pathology archive; follow-up data were updated through 31 December 2025, the prespecified follow-up cutoff date to ensure a minimum potential observation period for each case. Because mixed urothelial and small-cell carcinoma of the renal pelvis is exceedingly uncommon, a retrospective design was the only practical approach for assembling a clinicopathologically coherent series of sufficient size to permit paired immunohistochemical and exploratory survival analyses.

Potentially eligible cases were retrieved from the institutional pathology archive using diagnostic terms related to small-cell carcinoma, neuroendocrine carcinoma, renal pelvis, upper urinary tract, and mixed carcinoma. All available hematoxylin and eosin slides and corresponding clinical records were reviewed independently by two pathologists with subspecialty experience in genitourinary pathology. Cases were included when all of the following criteria were met: primary renal pelvic origin supported by clinicoradiological correlation; a histologically definite SmCC component meeting contemporary neuroendocrine carcinoma criteria (8, 9, 28); a concurrent morphologically distinct urothelial carcinoma component in the same specimen; and sufficient formalin-fixed paraffin-embedded (FFPE) tissue for the full immunohistochemical panel. Cases were excluded when the lesion represented pure SmCC without a demonstrable urothelial component, metastatic SmCC to the renal pelvis from another primary site, inadequate tissue for reliable immunohistochemistry, or prior neoadjuvant systemic therapy or radiotherapy that could materially alter morphology or antigen expression. Diagnostic disagreements were resolved at a multi-headed microscope until consensus was reached.

### Clinicopathological review and variable definition

Clinicopathological information was abstracted from pathology reports and electronic medical records using a predefined data collection form. Variables included age, sex, presenting manifestation, tumor laterality, maximum tumor size, pathological T and N classification, lymphovascular invasion, perineural invasion, surgical margin status, receipt of adjuvant chemotherapy, follow-up duration, and vital status at last contact. Pathological stage was assigned according to the American Joint Committee on Cancer (AJCC) eighth edition. Overall survival was defined as the interval from the date of surgery to death from any cause or to the date of last follow-up for censored observations. The proportions of the SmCC and UC components were estimated on review slides as percentages of total viable tumor area, with emphasis on the slide judged most representative of the overall tumor and cross-checking against additional available sections when needed to avoid overreliance on a focal field. Although this semi-quantitative estimate cannot replace digital whole-slide quantification, it reflects the manner in which mixed tumors are routinely assessed in diagnostic practice and permitted the neuroendocrine burden to be incorporated into the outcome analysis.

Histologically, the UC component was defined by conventional urothelial architecture and cytology, whereas the SmCC component was identified by small to intermediate cells with scant cytoplasm, hyperchromatic nuclei, finely granular chromatin, inconspicuous nucleoli, nuclear molding, brisk mitotic activity, and frequent necrosis (9, 28, 29). Particular attention was directed to transition zones between components, because one purpose of the study was to determine whether immunohistochemistry could clarify compartmental boundaries in morphologically ambiguous areas. For each case, the paired tumor compartments were evaluated separately rather than collapsed into a single global score.

### Immunohistochemistry and scoring

Immunohistochemistry was performed on 4-µm FFPE sections using the following antibody panel: INSM1, synaptophysin, chromogranin A, CD56, CK7, GATA3, Ki-67, and p53. Available assay information, staining controls, localization patterns, positivity thresholds, and scoring criteria are summarized in **Supplementary Table 1** to improve reproducibility. Because exact clone, dilution, staining-platform, and antigen-retrieval details were not uniformly retrievable from the retrospective archival records, this limitation is stated transparently in the **Supplementary Table** rather than inferred *post hoc*. The rationale for the panel was threefold: to document neuroendocrine differentiation, to confirm urothelial lineage, and to characterize biological aggressiveness through proliferative activity and p53 status (16–19, 24, 25). Positive and negative external controls were included in each staining run, and the paired tumor components on the same slide also served as internal contextual controls. INSM1 was scored only when unequivocal nuclear labeling was present. Synaptophysin and chromogranin A were interpreted as cytoplasmic stains, CD56 as membranous or membranocytoplasmic staining, CK7 and GATA3 according to their expected epithelial and nuclear patterns, and Ki-67 as the percentage of positive nuclei within hotspot areas. A threshold of at least 1% of tumor cells with positive staining was used to define marker positivity. Semi-quantitative expression for INSM1 and the conventional neuroendocrine markers was additionally recorded with a modified H-score, calculated as the sum of the products of staining intensity (0–3) and the percentage of cells at each intensity, yielding values from 0 to 300. p53 expression was categorized as aberrant when it demonstrated either diffuse strong overexpression or a complete-null pattern in the presence of intact internal control staining.

### Statistical analysis

The statistical plan was defined before formal analysis. Continuous variables were summarized as median with range or interquartile range, as appropriate, and categorical variables were summarized as counts and percentages. Distributional assumptions for continuous measurements were examined with the Shapiro-Wilk test. Because immunohistochemical comparisons were paired within the same case and several variables displayed non-Gaussian behavior, differences in marker positivity between INSM1 and each traditional neuroendocrine marker were assessed with the McNemar test, and paired quantitative comparisons, including the Ki-67 index between the SmCC and UC compartments, were analyzed with the Wilcoxon signed-rank test. Diagnostic indices for each marker were calculated against consensus histopathological compartment assignment as the reference standard. For this component-level analysis, each case contributed one positive compartment (SmCC) and one negative compartment (UC), permitting calculation of sensitivity, specificity, positive predictive value (PPV), and negative predictive value (NPV). Because this paired design structurally constrains PPV and specificity, these metrics were interpreted descriptively, whereas comparative emphasis was placed on sensitivity and discordant paired results. Exact binomial 95% confidence intervals were derived for sensitivity estimates, as appropriate for the small sample size.

**Figure 1:**
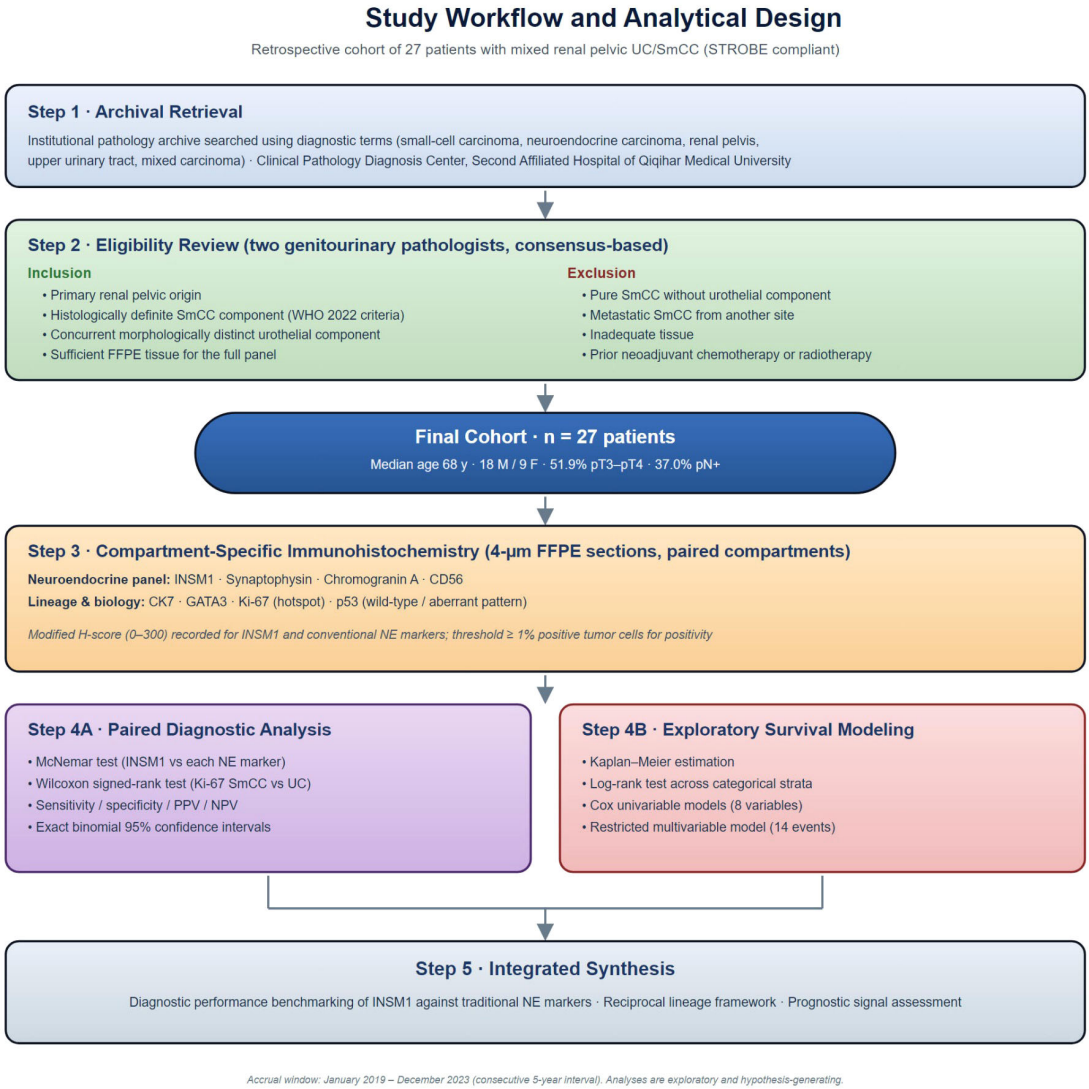
Study workflow and analytical design. The schematic summarizes case retrieval from the institutional pathology archive, independent slide re-review by two genitourinary pathologists, confirmation of mixed urothelial and small-cell histology against prespecified inclusion and exclusion criteria, compartment-specific immunohistochemical scoring, paired statistical comparisons, and exploratory Cox proportional hazards modeling.

Overall survival was explored with the Kaplan-Meier method and compared across categorical strata with the log-rank test. Cox proportional hazards regression was then used to estimate hazard ratios (HRs) and 95% confidence intervals (CIs) for prespecified clinicopathological and immunohistochemical variables. Univariable models included age, sex, pathological stage group, nodal status, lymphovascular invasion, SmCC proportion, adjuvant chemotherapy, and INSM1 H-score. INSM1 H-score was retained as a continuous prespecified exploratory variable; because of the small cohort and clustered upper-range values, formal nonlinear modeling was not attempted and the corresponding hazard ratio was interpreted cautiously. Because only 14 death events were observed, the multivariable model was deliberately restricted to a small set of clinically important covariates to limit overfitting, in accordance with current recommendations on events-per-variable constraints in survival modeling; specifically, pathological stage group, SmCC proportion greater than 50%, adjuvant chemotherapy, and INSM1 H-score were entered together. All tests were two-sided, and a P value less than 0.05 was considered statistically significant. No formal adjustment for multiple comparisons was applied because the study was exploratory and hypothesis-generating; accordingly, P values from pairwise marker comparisons were interpreted cautiously. The proportional hazards assumption was checked by inspection of log-minus-log plots and Schoenfeld residual diagnostics, without evidence of major violation, although the limited sample size reduces the power of these assessments. Statistical analyses were performed with SPSS version 26.0 and R version 4.3.2. The overall study workflow is summarized in **Figure 1**.

## Results

### Clinicopathological characteristics

Twenty-seven patients met the study criteria for mixed renal pelvic UC/SmCC. The cohort was composed predominantly of men (18 male and 9 female patients), and the median age at diagnosis was 68 years (range, 56–77 years). Hematuria was the most frequent presenting manifestation, occurring in 17 patients (63.0%), whereas flank pain and incidental radiological detection each accounted for 5 cases (18.5%). Tumor laterality was nearly balanced (14 left-sided and 13 right-sided tumors), and the median tumor size was 4.8 cm (interquartile range, 3.9–5.5 cm). Taken together, these baseline features describe a predominantly older cohort with clinically apparent disease rather than an incidentally discovered low-volume neoplasm.

The pathological distribution underscored the aggressive character of the series. Only 4 tumors (14.8%) were classified as pT1, whereas 9 (33.3%) were pT2, 9 (33.3%) were pT3, and 5 (18.5%) were pT4; accordingly, more than half of the cohort presented with pT3–pT4 disease. Lymph node metastasis was documented in 10 patients (37.0%), lymphovascular invasion in 13 (48.1%), perineural invasion in 6 (22.2%), and a positive surgical margin in 4 (14.8%). The median estimated proportion of the SmCC component was 58% (range, 15–95%), indicating that the neuroendocrine element was frequently not merely focal but biologically prominent. Seventeen patients (63.0%) received adjuvant chemotherapy, whereas 10 (37.0%) did not. Median follow-up was 19 months (range, 9–34 months). At last contact, 14 patients (51.9%) had died and 13 (48.1%) were alive/censored at last follow-up. All time-to-event analyses in the present study used overall survival, defined as death from any cause, and these outcome data further emphasize the unfavorable clinical trajectory of this mixed histology.

### Morphological features

**Figure 2:**
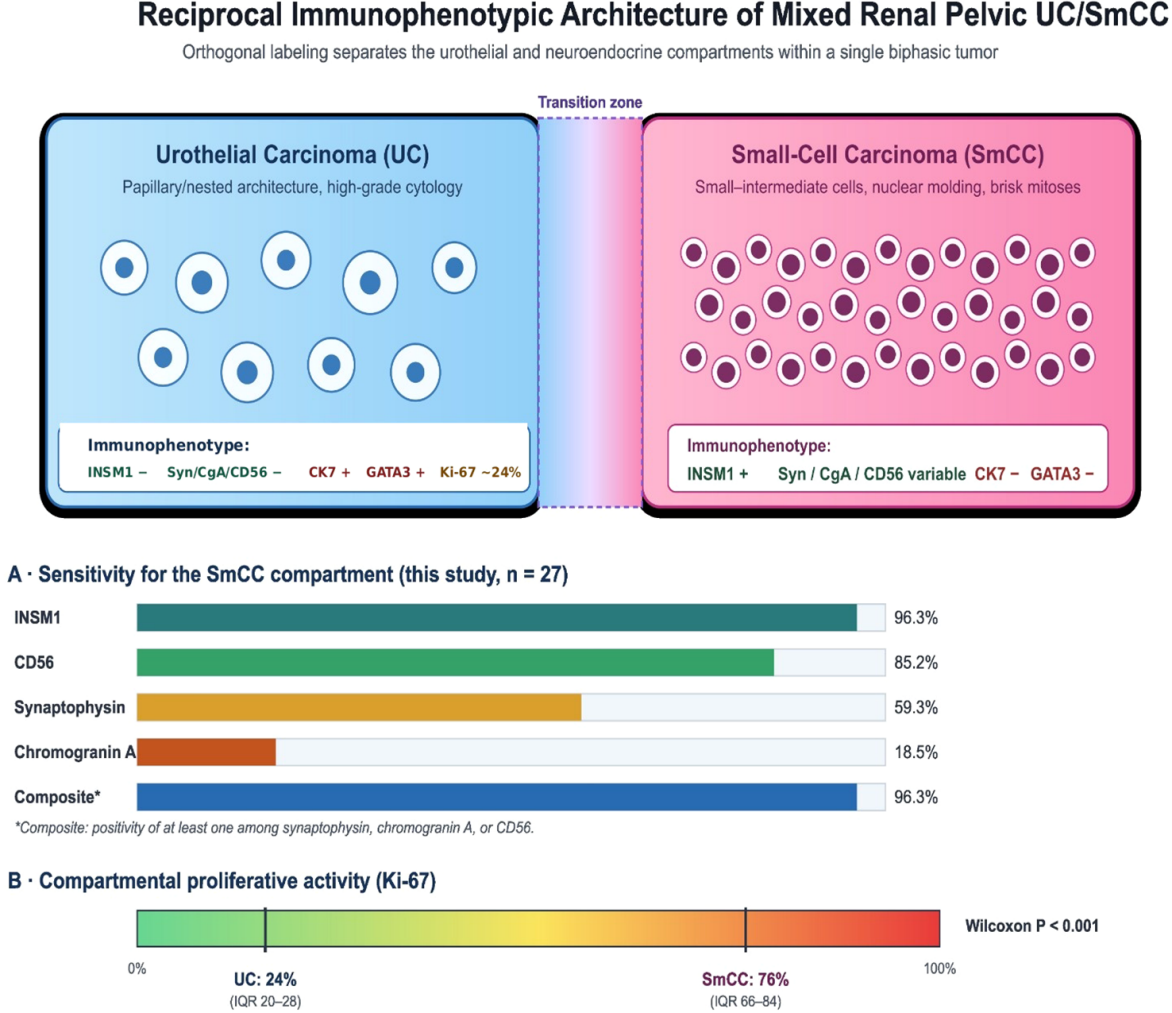
Schematic representation of the reciprocal immunophenotypic architecture of mixed renal pelvic UC/SmCC. The schematic illustrates the compartmental separation of urothelial carcinoma and small-cell carcinoma within a biphasic renal pelvic tumor. The urothelial carcinoma component is characterized by papillary or nested architecture, high-grade cytology, retained CK7 and GATA3 expression, absence of INSM1, negativity for traditional neuroendocrine markers, and a lower proliferative index. The small-cell carcinoma component is characterized by small to intermediate cells, nuclear molding, brisk mitotic activity, INSM1 nuclear positivity, variable expression of traditional neuroendocrine markers, loss of CK7 and GATA3, and a higher Ki-67 index. The central transition zone highlights the morphologically ambiguous interface in which an orthogonal immunohistochemical approach may assist compartmental delineation. This image is intended as a conceptual summary of the morphology-anchored immunophenotypic framework rather than as a representative photomicrographic panel.

On slide review, every case demonstrated a biphasic neoplasm composed of conventional UC and morphologically recognizable SmCC. The urothelial component retained the architectural and cytological features expected of high-grade UC, while the SmCC component displayed sheets, nests, or infiltrative aggregates of small to intermediate cells with scant cytoplasm, hyperchromatic nuclei, nuclear molding, brisk mitotic activity, and frequent necrosis. In several specimens the boundary between compartments was abrupt; in others, a transition zone was evident, particularly at the infiltrative front or along areas of extensive necrosis. These morphologically equivocal interfaces constituted precisely the settings in which the value of a robust immunohistochemical discriminator became most apparent. The reciprocal morphological and immunophenotypic architecture is summarized in **Figure 2**.

### Immunohistochemical performance of INSM1 and traditional markers

INSM1 demonstrated the highest single-marker sensitivity and clear compartment specificity for the SmCC compartment. Twenty-six of 27 SmCC components were positive, yielding a sensitivity of 96.3% (95% CI, 81.0–99.9%), while all 27 paired UC components were negative, producing a specificity of 100.0%. The positive predictive value and negative predictive value were 100.0% and 96.4%, respectively, and the median H-score in the SmCC compartment was 215 (interquartile range, 180–250). By comparison, synaptophysin was positive in 16 of 27 SmCC components (59.3%; 95% CI, 38.8–77.6%), chromogranin A in 5 of 27 (18.5%; 95% CI, 6.3–38.1%), and CD56 in 23 of 27 (85.2%; 95% CI, 66.3–95.8%). All three markers remained negative in the paired UC component, so their specificities were also 100.0%; however, their ability to identify the neuroendocrine compartment was considerably more variable. In paired McNemar testing, synaptophysin and chromogranin A were significantly less sensitive than INSM1 (P < 0.01 and P < 0.001, respectively), whereas CD56 did not differ significantly from INSM1 (P = 0.375). The corresponding discordant pair counts were 10 versus 0 for INSM1-positive/synaptophysin-negative versus INSM1-negative/synaptophysin-positive cases, 21 versus 0 for INSM1-positive/chromogranin A-negative versus INSM1-negative/chromogranin A-positive cases, and 4 versus 1 for INSM1-positive/CD56-negative versus INSM1-negative/CD56-positive cases. Of note, the conventional composite panel, defined as positivity of at least one among synaptophysin, chromogranin A, and CD56, achieved the same sensitivity as INSM1 alone (26 of 27, 96.3%), but required multiple stains rather than a single nuclear marker. This permissive any-positive definition was used to maximize sensitivity and should not be interpreted as equivalent to stricter multi-marker diagnostic algorithms. Because quantitative H-score emphasis differed across markers, cross-marker comparisons of staining intensity and extent should be interpreted cautiously. 

### Reciprocal lineage staining and proliferative activity

**Figure 3:**
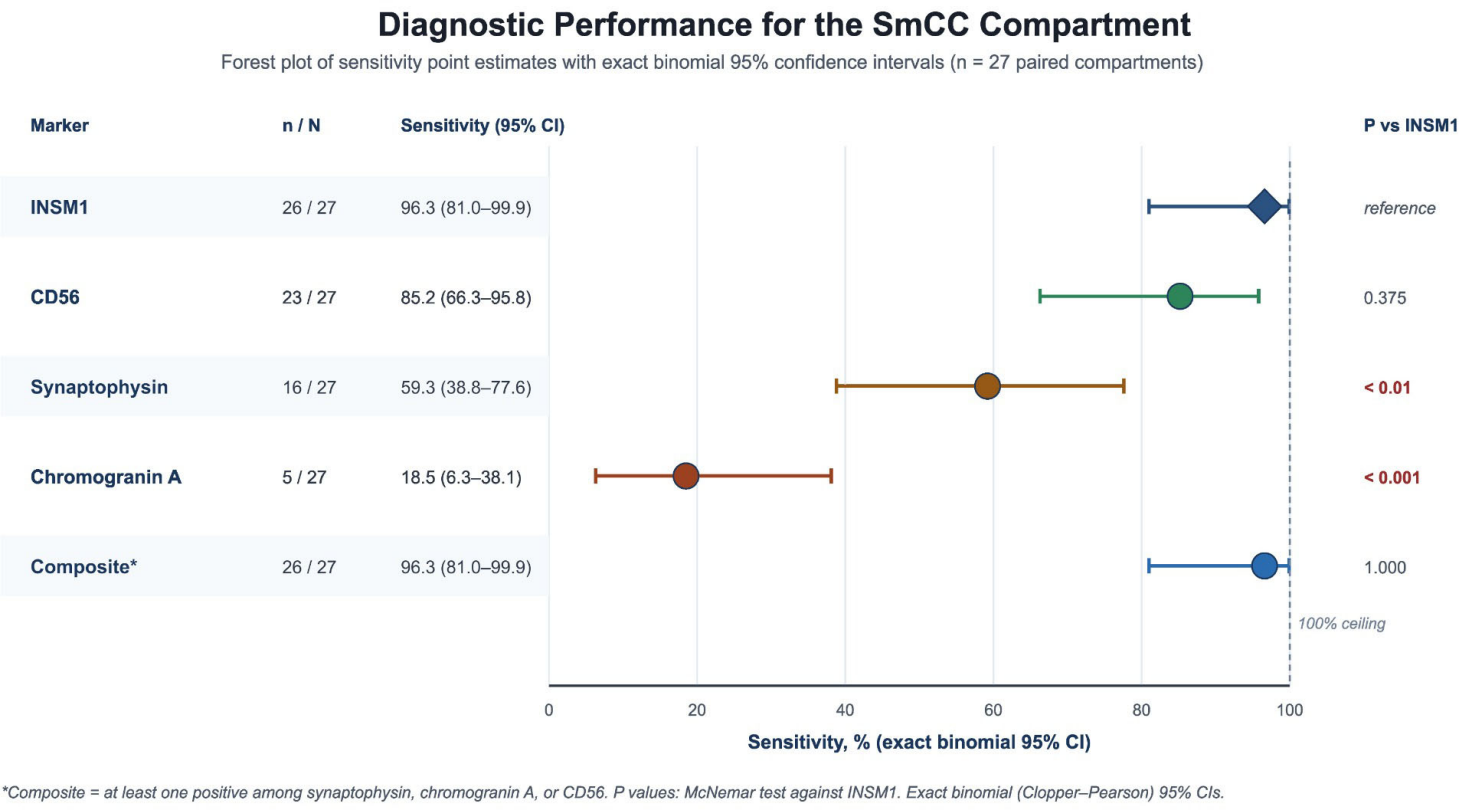
Sensitivity landscape of neuroendocrine markers for the SmCC component. Forest plot of point estimates with exact binomial 95% confidence intervals for sensitivity of INSM1, synaptophysin, chromogranin A, CD56, and the conventional composite panel, illustrating the high single-marker sensitivity of INSM1 and the similar sensitivity of INSM1 and the permissively defined three-marker composite panel.

Component delineation became even clearer when lineage-associated markers were considered together. CK7 and GATA3 were negative in all SmCC compartments but retained in all 27 UC compartments, generating a reciprocal staining pattern that complemented the INSM1 signal. In practical terms, the mixed tumors carried their own internal positive and negative controls: INSM1 highlighted neuroendocrine differentiation, whereas CK7 and GATA3 reinforced urothelial lineage. This reciprocity was particularly informative in zones where morphology was compressed, necrotic, or transitional. Biological divergence between the two compartments was further illustrated by proliferative activity. The median Ki-67 index was 76% (interquartile range, 66–84%) in SmCC and 24% (interquartile range, 20–28%) in UC, a highly significant paired difference (P < 0.001). Aberrant p53 expression was recorded in 14 of 27 SmCC components (51.9%) and in 14 of 27 paired UC components (51.9%). In a substantial subset, therefore, the two lineages displayed concordant disruption of p53 staining, a pattern compatible with a shared underlying molecular trajectory. The sensitivity estimates are summarized in **Figure 3**.

### Exploratory survival analysis

Exploratory survival analyses were consistent with the clinicopathological impression of an aggressive tumor driven, at least in part, by its neuroendocrine burden. On univariable Cox analysis, advanced pathological stage (pT3–pT4 vs pT1–pT2) was associated with a more than threefold increase in the hazard of death (HR, 3.42; 95% CI, 1.12–10.45; P = 0.031). Nodal metastasis carried a comparable adverse association (HR, 2.89; 95% CI, 1.03–8.12; P = 0.044). A predominant SmCC component, defined as greater than 50% of total tumor volume, was also unfavorable (HR, 3.67; 95% CI, 1.18–11.38; P = 0.025), as was an increasing INSM1 H-score when modeled per 10-unit increment (HR, 1.09; 95% CI, 1.01–1.18; P = 0.032). Lymphovascular invasion showed the expected harmful direction of effect but did not reach conventional statistical significance (HR, 2.41; 95% CI, 0.88–6.61; P = 0.087). By contrast, receipt of adjuvant chemotherapy was associated with reduced mortality risk on univariable analysis (HR, 0.31; 95% CI, 0.11–0.86; P = 0.024).

**Figure 4:**
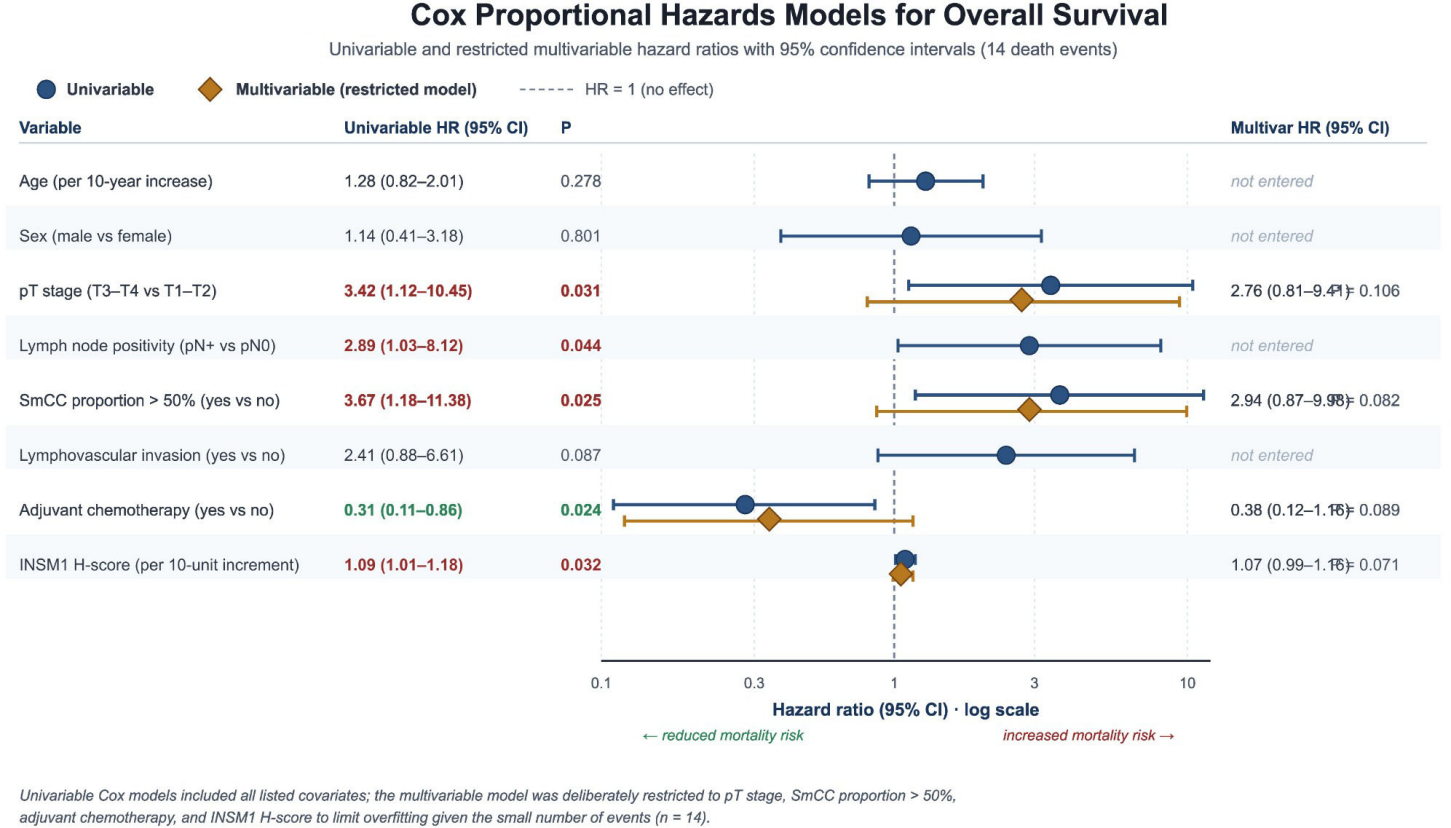
Forest plot of exploratory Cox proportional hazards models for overall survival. The plot presents univariable hazard ratios with 95% confidence intervals for the prespecified clinicopathological and immunohistochemical variables, with multivariable estimates superimposed for variables entered into the restricted adjusted model.

When these relationships were entered into a restricted multivariable model, effect sizes remained directionally consistent but became attenuated, a pattern that is unsurprising in a cohort of this size. Pathological stage group retained an HR of 2.76 (95% CI, 0.81–9.41; P = 0.106), SmCC proportion greater than 50% an HR of 2.94 (95% CI, 0.87–9.98; P = 0.082), adjuvant chemotherapy an HR of 0.38 (95% CI, 0.12–1.16; P = 0.089), and INSM1 H-score an HR of 1.07 per 10 units (95% CI, 0.99–1.16; P = 0.071). These estimates should be interpreted cautiously; their internal consistency is nevertheless notable, as variables reflecting greater anatomical extent, heavier neuroendocrine representation, and more intense INSM1 expression all trended toward worse outcome, whereas systemic treatment trended in the opposite direction. In a rare tumor for which mature evidence is difficult to generate, that internal coherence lends clinical plausibility to the observed associations even while definitive inference remains beyond the reach of the present cohort. The univariable and multivariable estimates are summarized in **Figure 4**.

## Discussion

The present study addresses a diagnostically important but rarely investigated problem: how best to identify and delineate the small-cell component in mixed urothelial carcinoma of the renal pelvis. The principal findings of this study can be summarized as follows. First, INSM1 was the most sensitive single marker for the SmCC compartment in this cohort and substantially outperformed synaptophysin and chromogranin A. Second, the combined use of INSM1 with CK7 and GATA3 provided a reciprocal immunophenotypic framework that cleanly separated neuroendocrine and urothelial lineages within the same tumor. Third, the clinicopathological data reinforced the impression that this neoplasm behaves aggressively, with outcome appearing to worsen as pathological extent and neuroendocrine burden increase.

The broader clinical context matters. UTUC represents only a small fraction of urothelial malignancy overall, yet it encompasses a range of variant morphologies that carry prognostic and therapeutic weight (1–7). Small-cell and other high-grade neuroendocrine carcinomas occupy the most ominous end of that spectrum (8, 9). Contemporary single-institution and multi-institutional series, together with recent systematic reviews, have consistently linked histological subtypes and divergent differentiation in UTUC to advanced pathological stage, nodal involvement, and inferior cancer-specific and overall survival, although much of this effect is mediated through stage (3, 5–7). Published experience with renal pelvic or upper urinary tract SmCC remains limited largely to case reports, pooled reviews, and small case series (10–15), and these reports consistently describe advanced presentation, rapid progression, and poor survival. The present cohort conforms closely to that pattern. More than half of the tumors were pT3–pT4, over one-third had nodal metastasis, nearly half showed lymphovascular invasion, and more than half of the patients had died at last follow-up. In other words, even before immunohistochemistry is considered, this is a clinicopathological setting in which diagnostic precision is likely to matter.

The diagnostic utility of INSM1 in our series is biologically plausible and aligns with the broader literature. In thoracic pathology, INSM1 has repeatedly demonstrated sensitivity equal or superior to that of traditional neuroendocrine markers for small-cell and related neoplasms (24, 25), and a recent systematic review and meta-analysis of 14 pulmonary studies (3,218 specimens) reported a pooled sensitivity of 0.86 and specificity of 0.97, with an area under the curve of 0.974, supporting its role as a reliable standalone marker (26). Genitourinary studies have extended that observation to urinary tract and other high-grade neuroendocrine carcinomas (20, 21), supporting the concept that INSM1 is not simply a lung-specific success story but a broadly useful marker of poorly differentiated neuroendocrine differentiation. Our data fit squarely within that trajectory. INSM1 identified 96.3% of SmCC compartments, whereas synaptophysin and chromogranin A showed substantially lower sensitivities. CD56 performed reasonably well, but its membranous staining pattern remains more susceptible to interpretive noise and lacks the compartmental clarity of a crisp nuclear signal. That the conventional three-marker panel matched the sensitivity of INSM1 alone is itself informative, but this finding should be interpreted in light of the permissive any-positive panel definition: in routine practice, a single well-performing nuclear marker may provide the same diagnostic yield that would otherwise require several stains, with corresponding savings in tissue, time, and interpretive complexity.

One of the most practically important observations in this study is that INSM1 was entirely absent from the paired urothelial component. This negative finding carries diagnostic value because it furnishes an internal within-case comparator. In mixed tumors, the pathologist is rarely asking only whether neuroendocrine differentiation exists somewhere in the case; the more difficult question is where it begins, how far it extends, and whether it accounts for the most aggressive-appearing area. When INSM1 is interpreted together with CK7 and GATA3, the answer becomes far more legible: the urothelial compartment remains highlighted by lineage-associated markers, while the neuroendocrine compartment is sharply labeled by a nuclear transcription factor. This orthogonal staining strategy is especially useful in transition zones, in crushed tissue, and in specimens with extensive necrosis. For these reasons, our findings suggest that INSM1 may be considered as an early companion stain in the immunohistochemical work-up of renal pelvic tumors in which small-cell morphology is suspected, rather than being reserved only for problematic or marker-negative cases.

The marked difference in Ki-67 between the two tumor compartments also warrants attention. The median proliferative index in SmCC was more than three times that of the urothelial component, reinforcing the impression that the neuroendocrine element represents the biologically dominant and most rapidly expanding fraction of the tumor. This observation is not merely descriptive. In mixed malignancies, treatment intensity is often driven less by the most prevalent component than by the most aggressive one, and the current findings support the view that even a morphologically admixed SmCC component may exert disproportionate influence on clinical behavior. The concordant aberrant p53 pattern observed in a substantial subset of paired components adds a further layer of interpretive interest. Although immunohistochemistry alone cannot establish clonality, the finding is consistent with integrated genomic studies of bladder small-cell carcinoma demonstrating shared alterations between urothelial and neuroendocrine elements, including near-universal TP53 inactivation, frequent RB1 pathway disruption, and evidence of urothelial-to-neural lineage plasticity (16–19). The conceptual model of a shared precursor followed by divergent differentiation therefore provides a more satisfactory explanation for many mixed tumors than the older notion of two unrelated lesions colliding within the renal pelvis.

The survival analysis should be interpreted as exploratory, but the findings are clinically coherent. Higher pathological stage, nodal disease, and a predominant SmCC component each behaved in the expected adverse direction. Adjuvant chemotherapy demonstrated a protective association on univariable analysis, a finding directionally consistent with the treatment logic usually applied to urinary tract small-cell carcinoma and with pooled upper urinary tract reports suggesting benefit from platinum-based regimens (14, 30), but this association may be affected by confounding by indication because treatment allocation was not randomized and performance status data were unavailable. The association between increasing INSM1 H-score and worse outcome is of particular interest. At a minimum, it suggests that the intensity and extent of neuroendocrine transcriptional commitment may track with aggressive biology. Whether INSM1 expression possesses independent prognostic value, however, cannot be settled by a cohort of this size. In the multivariable model, the effect estimates remained directionally stable but no longer crossed the conventional threshold of statistical significance, which is exactly the pattern one would expect when the number of outcome events limits statistical precision. The appropriate conclusion, therefore, is not that these factors have been disproved, but that they remain promising candidates for validation in larger collaborative datasets.

Several limitations warrant candid acknowledgment. The retrospective single-institution design introduces the usual risks of selection bias, incomplete clinical annotation, institutional and technical protocol bias, and variable follow-up. The rarity of mixed renal pelvic UC/SmCC necessarily constrained sample size, which in turn limited statistical power, model complexity, and precision of effect estimates. The reference standard was consensus histopathological compartment assignment rather than an independent gold standard, and no formal interobserver agreement metric was available. The study also remains dependent on immunohistochemical interpretation, and although consensus review reduces observer variability, it does not abolish it. In addition, detailed antibody clone, dilution, antigen retrieval, and staining platform information could not be uniformly retrieved from the archival records, which limits the reproducibility of the immunohistochemical findings, particularly the exact sensitivity estimates. Standardized intravesical recurrence and cystoscopic surveillance data were not consistently available, so bladder recurrence could not be analyzed without risk of ascertainment bias. In addition, the absence of comprehensive molecular profiling precludes direct testing of clonality and prevents integration of emerging genomic observations from bladder small-cell carcinoma into the present renal pelvic series. These caveats, however, do not negate the most actionable message of the study: within this morphology-anchored cohort, INSM1 showed consistently high sensitivity as a single marker for the SmCC compartment and was particularly useful for compartmental delineation in biphasic tumors.

From a practical standpoint, that message may be the most important one. Mixed renal pelvic UC/SmCC is sufficiently rare that many pathologists will encounter only a handful of cases, yet the consequences of missing the neuroendocrine component may be substantial. A marker that is sensitive, specific, and readily interpretable in compromised tissue can therefore deliver value disproportionate to the number of cases in which it is applied. Our findings are consistent with an approach in which morphology remains central, while INSM1 may be considered as an early companion stain and interpreted alongside urothelial lineage markers and proliferative assessment. Such an approach preserves diagnostic nuance while potentially rendering the work-up more efficient and reproducible.

## Conclusions

Mixed urothelial and small-cell carcinoma of the renal pelvis remains an uncommon but highly consequential malignancy. The present study indicates that INSM1 is a useful companion marker for recognition of the SmCC component and for clarification of compartmental boundaries within biphasic tumors. The survival findings should be viewed as exploratory and hypothesis-generating, but they suggest that pathological stage, nodal disease, and neuroendocrine burden may be clinically relevant in this setting. Larger multi-institutional studies, ideally coupled with molecular analysis, are needed to validate these observations and to define the prognostic significance of quantitative INSM1 expression more rigorously.

## Data Availability

The raw data supporting the conclusions of this article will be made available by the authors, without undue reservation.
