# Supplementary material for: Expression and prognostic significance of INSM1 compared with traditional neuroendocrine markers in mixed urothelial and small-cell carcinoma of the renal pelvis
Source: Front Oncol. 2026 Jul 20;16:1861172. doi: 10.3389/fonc.2026.1861172 (PMC13429432; doi:10.3389/fonc.2026.1861172)
Supplement: Supplementary file 1 [file Table1.docx]

**Table 1. Clinicopathological characteristics of the 27-patient cohort**

| **Variable** | **Value** |
| --- | --- |
| Number of patients | 27 |
| Age, years, median (range) | 68 (56-77) |
| Sex, male/female, n | 18/9 |
| Presenting manifestation, n (%) | Hematuria 17 (63.0); flank pain 5 (18.5); incidental detection 5 (18.5) |
| Tumor laterality, n (%) | Left 14 (51.9); right 13 (48.1) |
| Tumor size, cm, median (IQR) | 4.8 (3.9-5.5) |
| SmCC proportion, %, median (range) | 58 (15-95) |
| UC proportion, %, median (range) | 42 (5-85) |
| pT stage, n (%) | pT1 4 (14.8); pT2 9 (33.3); pT3 9 (33.3); pT4 5 (18.5) |
| pN stage, n (%) | pN0 17 (63.0); pN+ 10 (37.0) |
| Lymphovascular invasion, n (%) | Absent 14 (51.9); present 13 (48.1) |
| Perineural invasion, n (%) | Absent 21 (77.8); present 6 (22.2) |
| Surgical margin, n (%) | Negative 23 (85.2); positive 4 (14.8) |
| Adjuvant chemotherapy, n (%) | No 10 (37.0); yes 17 (63.0) |
| Follow-up, months, median (range) | 19 (9-34) |
| Vital status at last follow-up, n (%) | Dead 14 (51.9); alive/censored 13 (48.1) |

Continuous variables are presented as median (range) or median (interquartile range), as appropriate. Overall survival was defined as the interval from surgery to death from any cause or last follow-up for censored observations. IQR, interquartile range; SmCC, small-cell carcinoma; UC, urothelial carcinoma.
