# Supplementary material for: Expression and prognostic significance of INSM1 compared with traditional neuroendocrine markers in mixed urothelial and small-cell carcinoma of the renal pelvis
Source: Front Oncol. 2026 Jul 20;16:1861172. doi: 10.3389/fonc.2026.1861172 (PMC13429432; doi:10.3389/fonc.2026.1861172)
Supplement: Supplementary file 2 [file Table2.docx]

**Table 2. Immunohistochemical expression profiles and sensitivity estimates**

| **Marker** | **SmCC+ n/N (%)** | **UC+ n/N (%)** | **Sensitivity (95% CI), %** | **Specificity, %** | **PPV, %** | **NPV, %** | **H-score, median (IQR)** | **Discordant pairs vs INSM1*** | **P vs INSM1** |
| --- | --- | --- | --- | --- | --- | --- | --- | --- | --- |
| INSM1 | 26/27 (96.3) | 0/27 (0) | 96.3 (81.0-99.9) | 100.0 | 100.0 | 96.4 | 215 (180-250) | Reference | - |
| Synaptophysin | 16/27 (59.3) | 0/27 (0) | 59.3 (38.8-77.6) | 100.0 | 100.0 | 71.1 | - | 10; 0 | < 0.01 |
| Chromogranin A | 5/27 (18.5) | 0/27 (0) | 18.5 (6.3-38.1) | 100.0 | 100.0 | 55.1 | - | 21; 0 | < 0.001 |
| CD56 | 23/27 (85.2) | 0/27 (0) | 85.2 (66.3-95.8) | 100.0 | 100.0 | 87.1 | - | 4; 1 | 0.375 |
| Composite panel† | 26/27 (96.3) | 0/27 (0) | 96.3 (81.0-99.9) | 100.0 | 100.0 | 96.4 | - | 1; 1 | 1.000 |
| CK7 | 0/27 (0) | 27/27 (100) | - | - | - | - | - | - | - |
| GATA3 | 0/27 (0) | 27/27 (100) | - | - | - | - | - | - | - |
| Ki-67, median % (IQR) | 76 (66-84) | 24 (20-28) | - | - | - | - | - | - | < 0.001§ |
| p53 aberrant, n/N (%) | 14/27 (51.9) | 14/27 (51.9) | - | - | - | - | - | - | - |

* Discordant pairs are shown as INSM1-positive/marker-negative; INSM1-negative/marker-positive case counts in the SmCC compartment.

† Composite panel was considered positive when at least one among synaptophysin, chromogranin A, or CD56 was positive. This permissive any-positive definition favors sensitivity and should be interpreted descriptively.

P values compare paired SmCC positivity rates with INSM1 by the McNemar test. § Wilcoxon signed-rank test comparing Ki-67 index between SmCC and UC compartments.

Because each case contributed one positive SmCC compartment and one negative UC compartment, specificity and PPV are structurally constrained by the paired design and should be interpreted descriptively. H-score emphasis was limited to INSM1; cross-marker quantitative intensity/extent comparisons were not used as primary comparative endpoints.

CI, confidence interval; IQR, interquartile range; NPV, negative predictive value; PPV, positive predictive value; SmCC, small-cell carcinoma; UC, urothelial carcinoma.
