# Supplementary material for: Expression and prognostic significance of INSM1 compared with traditional neuroendocrine markers in mixed urothelial and small-cell carcinoma of the renal pelvis
Source: Front Oncol. 2026 Jul 20;16:1861172. doi: 10.3389/fonc.2026.1861172 (PMC13429432; doi:10.3389/fonc.2026.1861172)
Supplement: Supplementary file 3 [file Table3.docx]

**Table 3. Univariable and restricted multivariable Cox proportional hazards models for overall survival**

| **Variable** | **Univariable HR (95% CI)** | **P value** | **Multivariable HR (95% CI)** | **P value** |
| --- | --- | --- | --- | --- |
| Age (per 10-year increase) | 1.28 (0.82-2.01) | 0.278 | - | - |
| Sex (male vs female) | 1.14 (0.41-3.18) | 0.801 | - | - |
| pT stage (T3-T4 vs T1-T2) | 3.42 (1.12-10.45) | 0.031 | 2.76 (0.81-9.41) | 0.106 |
| Lymph node positivity (pN+ vs pN0) | 2.89 (1.03-8.12) | 0.044 | - | - |
| SmCC proportion > 50% (yes vs no) | 3.67 (1.18-11.38) | 0.025 | 2.94 (0.87-9.98) | 0.082 |
| Lymphovascular invasion (yes vs no) | 2.41 (0.88-6.61) | 0.087 | - | - |
| Adjuvant chemotherapy (yes vs no) | 0.31 (0.11-0.86) | 0.024 | 0.38 (0.12-1.16) | 0.089 |
| INSM1 H-score (per 10-unit increment) | 1.09 (1.01-1.18) | 0.032 | 1.07 (0.99-1.16) | 0.071 |

Overall survival was defined as death from any cause. The multivariable model was intentionally restricted to a small number of clinically relevant variables to reduce overfitting in a cohort with 14 death events; estimates should be interpreted as exploratory and hypothesis-generating. CI, confidence interval; HR, hazard ratio; SmCC, small-cell carcinoma.
